# Crime and Criminals

**Published:** 1889-08-10

**Authors:** 


### Crime and Criminals.

Crime is one of the subjects on which it is most difficult to get any opinion worth listening to. Those who speak or write on the subject seem to be divided into two classes—the stern jurists who desire only to condemn the criminal to penal servitude, with hard labour and the "cat," for all offences for which he cannot be hanged; and the sentimentalists, who "poor dear" the murderer, who—becoming scientific—trace the forger's decline and fall from the writing prize his great grandfather won at school, and who, plunging into rhetoric, declare, truthfully enough, that many worse people go about at large than those who fill our prisons. On the errors of heart and head evinced by both classes we need not enter, but it is worth while to point out the failure in logical precision that characterises both. The first confound the criminal with the crime, the second hold vice and crime to be identical; vice, in which the sinner directly injures himself, and crime, whereby he primarily hurts other people.

It is, therefore, the more refreshing to come across anything so sane, just, and practical as the pamphlet published by Mr. Frederick Howard Wines, under the title of "Crime, the Convict, and the Prison." Mr. Wines has devoted time, energy, and intelligence to the question of charity—that charity which developes self-respect instead of lessening its action, and seeks to make even the worst of men a decent member of society. To do this is not, in all cases, humanly possible. It is better to recognise this at once. By so doing, we do not lessen the chance given to the sinner to leave his evil ways; but we feel justified in taking such precautions as will prevent his evil nature from contaminating others who, though they have fallen into evil, are not yet acclimatised to it.

With regard to the confirmed criminal, it may be doubted if our system of punishment does any good whatsoever. Those he has wronged are safe from him only so long as he is shut up in prison. To form a real safeguard to society he should be imprisoned for life, if the swift and ready method of capital punishment be dispensed with. We do not now propose to discuss the case for hanging. It may be the worst use a man can be put to, or the simplest solution of a difficult problem. But all legal penalties, other than death, ought to be reformatory in intention. If they are not, they are, from a social point of view, harmful. It is commonly said, and with some degree of truth, that a "gaol-bird" comes out of prison a worse criminal than he went in. He has learned, during his period of confinement, to turn a crank, to walk a treadmill, or to pick oakum—industries which are not in great demand in the outer world. For mental exercise he has had brooding over the blunder which led to his conviction, planning new crimes of a superior order which shall escape undetected, and longing for vengeance on the persons who brought him to justice. Such things do not make for good citizenship, and the instinct which makes employers of labour refuse men who have been "in trouble," though it may be cruel, is not wholly unjust. One who has been in prison is likely to be a worse man than one who has not, not merely because of his crime, but because of the effects of his punishment. The primary intention of punishment was simply vindictive; it was meant to avenge one injury by inflicting another, and the spirit in which justice—to be accurate, we should say vengance—was administered in ancient times still survives in our own day. But if, indeed, we have no intention of trying to reform the prisoner when we send him to prison, we had better take his life at once, and leave him to the judgment of God.

Our prisons are better than they were. They are no longer breeding places for disease; prisoners are no longer in danger of starvation; a certain propriety of conduct is enforced. But to say that they can be regarded with honest satisfaction, as institutions which actively contribute to the public welfare, would be rash. Our reformatories are a step in the right direction, though they are not yet perfect; but all our prisons should be based on the principle of the reformatory.

It may be said that this is impossible with adults, and that while a child is sent to a reformatory for years, a man goes to prison only for a few weeks or months, in which time little good can be done. This difficulty has been obviated in some of the American States by what is called the "indeterminate sentence." Strictly speaking, this would mean a possible life sentence for every conviction, but modified in action the result is this: that every prisoner is sentenced for "the maximum term provided by law for the crime for which he is convicted." This apparent severity is modified by the fact that the prisoner himself can reduce his term of imprisonment by his own good conduct; an appeal is made to his better nature by the chance of regaining his liberty before the expiration of the period for which he is sentenced, through his own exertions.

The system is that of a school, which, however rigid, is better than that of the prison pure and simple. Marks are given for good conduct, for industry, and for attention in the prison school. There are three grades of prisoners, and a new convict, on entering the gaol, is placed in the middle grade, to be degraded or promoted, according to his conduct. In this grade they are not labelled with the "broad arrow" or its equivalent. They wear a "citizen's suit," they march by twos, have tea and coffee, library books, and gas to study by at night; they have a chair in their cells, sheets, slippers, and brushes; are allowed to receive letters once a week, and to write once a month. If, by laziness or unruly conduct, they forfeit these privileges they are degraded to the third grade, where they are dressed in red, and march in lock-step, and are deprived of tea and coffee, books, gas, and the privilege of writing letters. Their cells, also, are more poorly furnished than those in the other grades.

If, on the other hand, they gain the marks obtainable by good conduct, industry, and attention, they are promoted to the first grade and its extra privileges. This cannot be done in less than six months. The maximum number of marks given is settled by the authorities. It is, of course, a purely arbitrary matter, and the absolute number affixed does not matter so long as a proper proportion is maintained. At the prison in Elmira, in the State of New York, where the principle of the indeterminate sentence is carried out, the maximum number for each of the three points noted above is three, making nine marks in all, which a prisoner desirous of improving his position can gain in a day. If for six months in succession after entering the prison he "earns his nines," as it is phrased, he is put into the first grade, where the men take their meals in a separate dining-room, and are allowed to converse at table, write letters once a week, have gas in their cells an hour later than others, and, among other things, are eligible for appointments of trust in the prison.

If during the time they are in the first grade they "earn their nines" for six more months, they become candidates for successful liberation. Their obtaining this is left to the discretion of the managers of the prison. The law runs thus: "When it appears to the said managers that there is a strong or reasonable probability that any prisoner will live and remain at liberty without violating the law, and that his release is not incompatible with the welfare of society, then they shall issue to such prisoner an absolute release from imprisonment." The discretion and authority thus vested in the manager of a prison make it necessary that he should be a man of superior qualities—just, conscientious, and discriminating; and in their degree of responsibility these same qualities are required in his subordinates. A mere "turnkey" is worse than useless; to be worthy of his post the gaoler must no longer be a character whom society regards with very little less aversion than the criminals under his charge.

But, given the right men as warders and superintendents, the method of the indeterminate sentence, with its wholesome discipline of work for the avowed object of obtaining freedom, gives a ground for regarding prisons as agencies in social improvement. Work may not be salvation, though it makes for it, but we do not need to go so far as Dr. Watts to be convinced that idleness is damnation. By work we mean, let it be premised, something that exercises hand and brain, and leads to a useful result; not cranks and treadmills, or any other malicious invention for the development of unprofitable fatigue. Many convicts come into prison ignorant of all arts save those of lying, theft, and burglary; if they return to the world when their term is up uninstructed in anything better, they must inevitably return to crime. If they go out furnished with the knowledge of a trade, they have at least a chance of becoming honest citizens. For those prisoners of another class, educated men who have fallen under the stress of sudden temptation, the discipline is equally valuable. The chance of improving their position even in prison, the hope of redeeming their liberty by their own efforts, helps to raise their broken self-respect, and sends them out into the world more fit than they would otherwise be to struggle for a place in it. The lot of the ex-prisoner will be a hard one till the end of time. Human nature must prove more kind and generous than we yet know it to be before the stain on his past is allowed to cast no shadow on his future. But this plan of the indeterminate sentence, with its incentive of hope and its discipline of work, is the best that has yet been discovered for fitting him for the struggle that lies before him.

